# Comparative analysis of chloroplast genomes of endangered heterostylous species *Primula wilsonii* and its closely related species

**DOI:** 10.1002/ece3.9730

**Published:** 2023-01-16

**Authors:** YanPing Xie, GangGang Yang, Chan Zhang, XingWang Zhang, XianFeng Jiang

**Affiliations:** ^1^ School of Life Sciences Huaibei Normal University Huaibei China; ^2^ School of Life Sciences Henan Normal University Xinxiang China; ^3^ Yunnan Key Laboratory of Biodiversity and Ecological Security of Gaoligong Mountain Kunming China; ^4^ College of Agriculture and Bioscience Dali University Dali China

**Keywords:** Chinese *Primula*, phylogeny, plant species with extremely small populations, plastid genome, sequence divergences

## Abstract

*Primula*, well known for its heterostyly, is the largest genus in the family Primulaceae with more than 500 species. The considerable species number has introduced a huge challenge for taxonomy. The phylogenetic relationships among *Primula* still maintain unresolved due to frequent hybridization and introgression between closely related species. In this study, we sequenced and assembled the complete chloroplast genomes of *Primula wilsonii* Dunn, which is a PSESP (plant species with extremely small populations), using Illumina sequencing and compared its genomic sequences with those of four related *Primula* species. The chloroplast genomes of *Primula* species were similar in the basic structure, gene order, and GC content. The detected 38 SSRs (simple sequence repeats) loci and 17 hypervariable regions had many similarities in *P. wilsonii*, *P. anisodora*, *P. miyabeana*, and *P. poissonii*, but showed a significant difference compared with those in *P. secundiflora*. Slight variations were observed among *Primula* chloroplast genomes, in consideration of the relatively stable patterns of IR (inverted repeats) contraction and expansion. Phylogenetic analysis based on chloroplast genomes and protein‐coding genes confirmed three major clades in Chinese *Primula*, but the infrageneric sections were not in accordance with morphological traits. The *P. poissonii* complex was confirmed here and *P. anisodora* was the most closely related species to *P. wilsonii*. Overall, the chloroplast genome sequences provided useful genetic and evolutionary information for phylogeny and population genetics on Chinese *Primula* species.

## Graphical abstract

The study concentrated on the comparison of chloroplast genomes between a PSESP (plant species with extremely small populations) species and its closely related species in the genus *Primula*. The polymorphic repeat sequences were detected which could be used for further studies of *Primula* species. Furthermore, the phylogenetic relationships of Chinese *Primula* were conducted using chloroplast sequences.

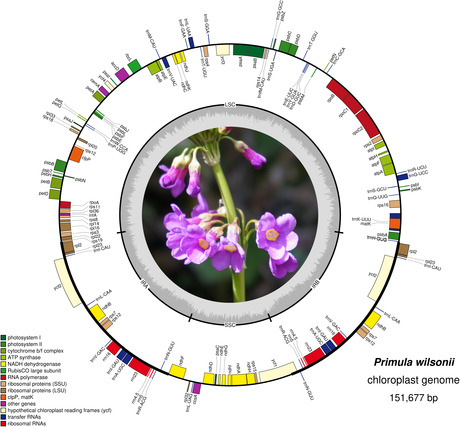

## 1 INTRODUCTION

*Primula* is famous for its mating with the heterostyly system, high ornamental value, and richness species. This group comprises about 500 species, with more than 300 species distributed in China, particularly clustering in the Himalaya–Hengduan Mountains (Richards et al., 2003). *Primula* is reputed to be one of the great garden plant genera throughout the world owing to the widely grown and long flowering period (Richards, 2002; Yan et al., 2010). The taxonomic study of *Primula* has been revised many times according to morphological characters. The infrageneric system with a total of 31 sections of *Primula* was initially proposed by Smith and Fletcher (Smith & Fletcher, 1947). Later, a revised system with seven subgenera was proposed that considered some putative reticulate evolutionary relationships (Wendelbo, 1961). Then, a six‐subgenera system was amended in *Primula* by Richards (Richards et al., 2003), whereas the subgenus concept was not adopted in the delimitation of the Chinese *Primula*: a total of 24 sections were delimited (Hu, 1990). Moreover, many key diagnostic traits, such as the shape of calyx, are slightly different and nonquantitative. The frequent natural hybridization and gene introgression also confuse species boundaries in this genus (De Vos et al., 2014; Guggisberg et al., 2009; Ma et al., 2014; Xie, Zhao, et al., 2017; Xie, Zhu, et al., 2017). Although an increasing number of phylogenetic constructions have been carried out previously and have greatly advanced our understanding of the evolution of *Primula*, the phylogenetic relationships within the genus *Primula* species have remained incompletely resolved.

*Primula wilsonii* Dunn is a perennial herb in Sect. *Proliferae* Pax of *Primula* (Primulaceae). The most closely related species of *P. wilsonii* are *P. miyabeana*, *P. poissonii*, and *P. anisodora*, and these species are clustered in a well‐supported clade based on phylogeny construction using *rbcL* + *matK* + *ITS* markers (Yan et al., 2015). *P. wilsonii* sporadically scatters in limited areas of Sichuan and Yunnan provinces, while the other widespread Primulas are common in the fields and gardens. Considering the limited distribution areas and small size of populations in the fields, *P. wilsonii* was identified as a PSESP (plant species with extremely small populations) and eagerly in need of protection (Sun et al., 2019).

Chloroplasts are the photosynthetic organelles in plant cells, with highly conserved genomes that are inherited maternally in major angiosperms (Daniell, 2007; Daniell et al., 2016; Moore et al., 2010). The quadripartite structure of the angiosperm chloroplast genome consists of a large single copy (LSC) region and a small single copy (SSC) region, separated by two inverted repeats (IRs) (Wang et al., 2016). Up till now, taking advantage of the development of high‐throughput sequencing technology, more than 7000 chloroplast genomes of land plants have been publicly known (Kirill et al., 2021) since the publication of the first plastid genome sequences for *Nicotiana tabacum* and *Marchantia polymorpha* (Ohyama et al., 1986; Shinozaki et al., 1986). Additionally, the lack of recombination and slow evolutionary rate as compared with mitochondrial and nuclear genomes make it suitable for phylogeny at genus and family level, species barcoding, population genetics and the conservation of endangered species (Daniell et al., 2016; Henriquez et al., 2014; Nguyen et al., 2017; Palmer et al., 1988; Wambugu et al., 2015; Wang et al., 2016; Zhai et al., 2019). And the comparative analysis of chloroplast genomes among closely related species and phylogenetic analysis in *Primula* provided insights into the evolutionary history (Li, 2022; Ren et al., 2018; Xu et al., 2020). Therefore, we determine the complete chloroplast genomes of a PSESP species in this genus (Xie et al., 2021). In addition, we explore simple sequence repeats (SSRs) loci and identify highly variable regions by comparing the genome contents and structures between the PSESP species *P. wilsonii* and its widely spread closely related species. Here, we aim to: (1) investigate the molecular structures of the chloroplast genomes of *P. wilsonii*, (2) examine the variations of SSRs and highly divergent regions that could be used as specific DNA markers for *P. wilsonii* and its close relatives, (3) evaluate the evolution of several *Primula* chloroplast genomes, and (4) facilitate the systematics of the Chinese *Primula* species.

## 2 MATERIALS AND METHODS

### 2.1 Plant materials, DNA extraction, and sequencing

The plant materials used in this study were collected from Wuxuhai, Sichuan Province, China (29.16 N, 101.41 E). The voucher specimen (voucher accession number XYP202007016) was deposited in the Key Laboratory of Plant Resource and Biology at Huaibei Normal University. Total genomic DNA was extracted from silica‐dried leaves with a modified cetyltrimethylammonium bromide (CTAB) method (Doyle & Doyle, 1987). The quality and quantity of the DNA samples were determined using agarose gel electrophoresis and the Qubit dsDNA BR assay (Life Technologies). The qualified PCR‐amplified library was sequenced with the Illumina NovaSeq Tenplatform (Nanjing Genepioneer Biotechnologies Inc.).

### 2.2 Genome assembly, annotation, and analysis

The low‐quality reads were assessed and filtered using FastQC (https://www.bioinformatics.babraham.ac.uk/projects/fastqc/) and Trimmomatic v 0.36 to obtain high‐quality clean data (Bolger et al., 2014). The chloroplast genome sequence of *P. wilsonii* was assembled by a de nova method using SPAdes Genome Assembler v3.10.1 (Bankevich et al., 2012). The k‐mers were set to 55, 87 and 121 to achieve optimal assembly. The whole chloroplast genomes were assembled using high‐coverage and long‐sequenced contigs. Then, the SSPACE v2.0 was used to construct the scaffold of the chloroplast genomes (Boetzer et al., 2011) and Gapfiller v2.1.1 was used to fill the gaps (Boetzer & Pirovano, 2012). Finally, Bowtie2 v2.2.4 was used to validate the genome assembly by mapping the initial reads to the assembled genome sequence (Langmead & Salzberg, 2012). The chloroplast genomes were annotated in two ways. Prodigal v2.6.3 (Hyatt et al., 2010), Hmmer v3.1b2 (Wu et al., 2011), and ARAGORN v1.2.38 (Laslett & Canback, 2004) were used to detect the protein‐coding sequences (CDS), Ribosomal RNA (rRNA), and Transfer RNA (tRNA), respectively. Furthermore, BLAST tools (National Center for Biotechnology Information, NCBI) were used to check the annotation, followed by manual correction through comparison with the other closely related chloroplast genomes. The circular chloroplast genome map of *P. wilsonii* was then generated using OGDraw (Lohse et al., 2013). The repeating sequences including forward, reverse, complement, and palindrome repeats were identified using the online REPuter program (Kurtz et al., 2001), with three for Hamming distance and 30 for minimal repeat size. SSRs were detected using MISA software (https://webblast.ipk‐gatersleben.de/misa/; Beier et al., 2017). Thresholds for a minimum number of repeat units were set as follows: 10 for mononucleotide repeat units; 5 for di‐; 4 for tri‐; and 3 for tetra‐, penta‐, or hexa‐nucleotide repeat units.

### 2.3 Comparative plastome analysis

Gene distribution and the percentage of sequence identity were compared and visualized using mVISTA software (Frazer et al., 2004) with the LAGAN mode (Brudno et al., 2003) in chloroplast genomes of five *Primula* species, with *P. secundiflora*, *P. poissonii*, *P. anisodora*, and *P. miyabeana* selected as close relatives of *P. wilsonii*. All the chloroplast genomes were obtained from NCBI, except for *P. wilsonii*. The annotation of *P. wilsonii* served as a reference. Nucleotide variability values (Pi) were calculated using the same alignment. Nucleotide diversity was detected by sliding window analysis conducted in DnaSP v.6.11.01 with a step size of 200 bp and window length of 600 bp (Rozas et al., 2017). The expansion or contraction of the inverted repeat (IR) regions in the chloroplast genomes of the five related *Primula* species were investigated and visualized using IRscope program (Amiryousefi et al., 2018).

### 2.4 Phylogenetic analyses

To investigate the phylogenetic relationship in the sections of Chinese *Primula* and the resolution ability of chloroplast genomes, phylogenetic analysis was performed for the 60 species representing 20/24 sections of Chinese *Primula* based on two datasets: the complete chloroplast genomes and 66 shared protein‐coding genes. The targeted 66 protein‐coding sequences were extracted and concatenated using Geneious Prime® 2020.1.1 (Kearse et al., 2012). Two outgroup species (*Andorsace bulleyana* and *Ardisia solanacea*) were sampled from a closely related genus *Andorsace* and a more distantly related genus *Ardisia* of the Primulaceae in the sense of phylogenetic relationships (Chen et al., 2016). The sequences were firstly aligned by MAFFT v7.307 (Katoh & Standley, 2013). jModelTest 2.0 was used to find the best nucleotide‐substitution model and was determined according to Akaike's information criterion (AIC) before phylogenetic construction (Darriba et al., 2012). Trees were then constructed using the maximum likelihood (ML) method by online RaxML BlackBox software (Stamatakis et al., 2008) and Bayesian inference (BI) method using MrBayes (Ronquist & Huelsenbeck, 2003). ML was implemented starting from random trees, using 1000 rapid bootstrap inferences with a General Time Reversible (GTR) nucleotide‐substitution model as suggested. The BI analysis was performed on the condition that Markov chain Monte Carlo calculation is set to 2,000,000 generations and sampled every 1000 generations. The remaining 1500 trees were used to generate the consensus tree after the first 25% of the generations were discarded as a burn‐in. The final phylogenetic results were viewed by using FigTree v.1.6.1.

## 3 RESULTS

### 3.1 Chloroplast genomes features

**FIGURE 1:**
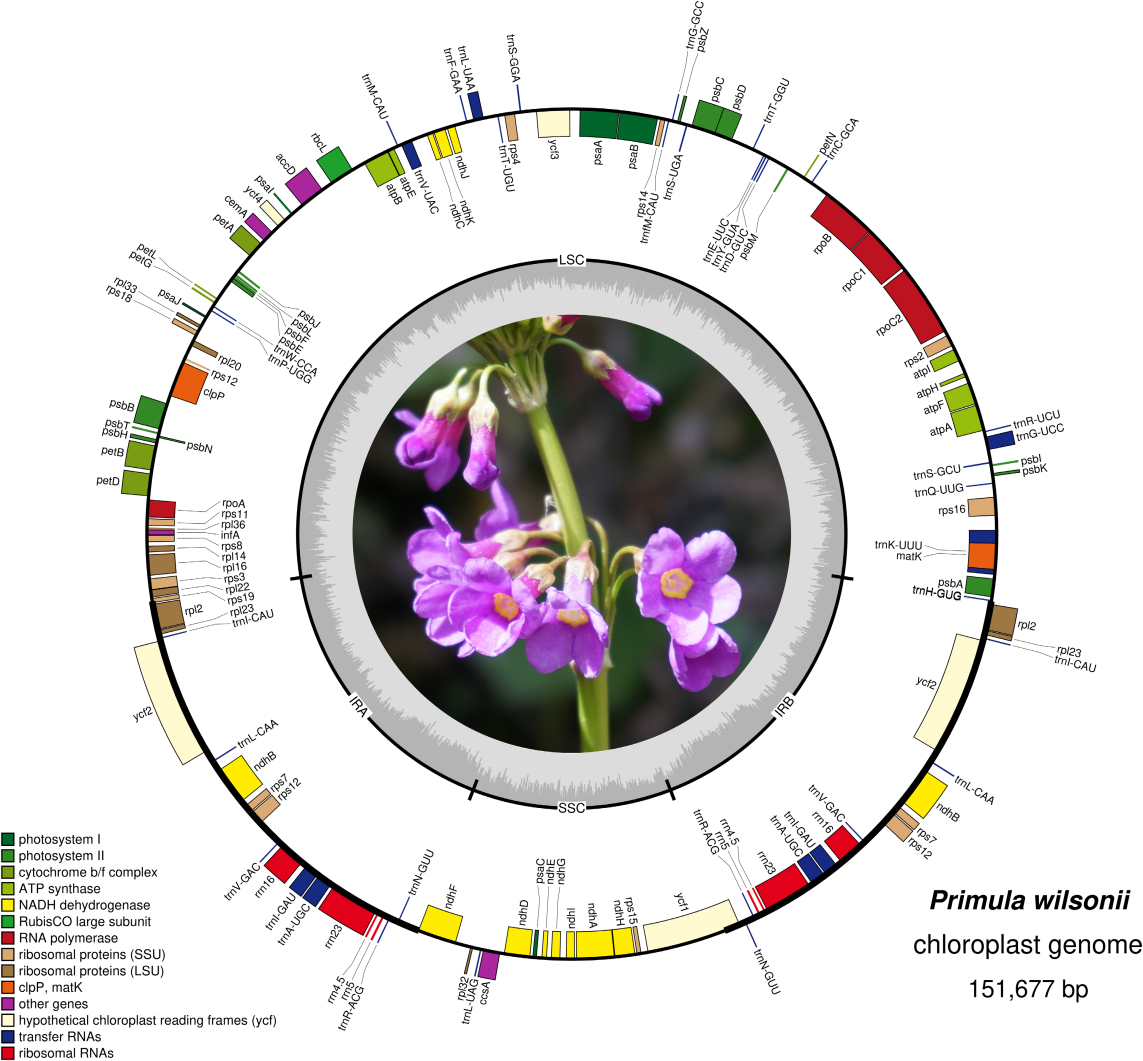
Circular gene map of the chloroplast genome of *Primula wilsonii*. The thick lines indicate the inverted repeat regions (IRa and IRb) that separate the genome into small (SSC) and large (LSC) single copy regions. Genes on the inside of the map are transcribed clockwise, while those on the outside are transcribed counterclockwise. Genes grouped by different functions are shown in different colors.

Among the 23,651,814 paired‐end clean reads generated by Illumina sequencing, 604,525 reads were mapped to the final assembly. Based on these data, we obtained a high‐quality plastome of *P. wilsonii*, with average coverage depth of 1249.46 ×. The assembled chloroplast genome of *P. wilsonii* was 151,677 bp in length, exhibiting a typical circular chloroplast structure like most angiosperms. As shown in Figure 1, it contains a LSC region of 83,510 bp, a SSC region of 17,765 bp, and a pair of inverted repeats (IRa and IRb) of 25,201 bp. Overall, the GC content was 36.99%. We found an uneven distribution of GC content across the regions of the genomes, which were 34.89%, 30.18%, and 42.87% for the LSC, SSC, and IR regions, respectively. The chloroplast genome structures, the size of each region, and the GC contents of the five chloroplast genomes of *Primula* species were compared and are shown in Table 1. The genome features were nearly identical in the five *Primula* Chloroplast genomes.

**TABLE 1:** Comparison of general features of *Primula* chloroplast genomes

Genome features	*P. wilsonii*	*P. anisodora*	*P. miyabeana*	*P. poissonii*	*P. secundiflora*
GenBank number	MW442886	NC053578	MT317303	NC024543	MT317261
Genome size (bp)	151,677	151,674	151,706	151,664	151,583
LSC length (bp)	83,510	83,498	83,520	83,444	83,362
SSC length (bp)	17,765	17,772	17,747	17,822	17,839
IR length (bp)	25,210	25,202	25,219	25,199	25,191
Total GC content (%)	36.99	37.02	37.00	37.02	36.98
Total genes	113	113	112	115	112
Protein‐coding genes	79	80	79	81	79
tRNA genes	30	29	29	30	29
rRNA genes	4	4	4	4	4

Regardless of the duplicate genes, the chloroplast genome of *P. wilsonii* contained 113 genes, including 79 protein‐coding genes, 30 tRNA genes, and four rRNA genes. Among them, six protein‐coding genes, seven tRNAs, and all the four rRNAs were completely duplicated within IRs (Figure 1). The *rps12* gene was found to be trans‐spliced in chloroplast genomes. It had two duplicates in IRs and one of its exons is located in the LSC region (Figure 1). A total of 18 genes, including 12 protein‐coding genes and six tRNA genes, had introns. It was noted that 16 genes among them had only one intron while the other two genes had two introns. The comparisons between the genome of *P. wilsonii* and the other *Primula* chloroplasts are shown in Table 1. The results indicated that the gene contents were similar among the *Primula* species, excluding the loss of the *ycf15* gene in the *P. wilsonii* chloroplast genome. The chloroplast genomes of *P. poissonii* and *P. wilsonii* possessed the *infA* gene, whereas the others did not have it. Moreover, the *trnG*‐GCC sequence only appeared in the chloroplast genome of *P. wilsonii* (Table 2).

**TABLE 2:** Lists of genomic genes for *Primula wilsonii*, *P. anisodora*, *P. miyabeana*, *P. poissonii*, and *P. secundiflora*

Group of genes	*P. wilsonii* MW442886	*P. anisodora* NC053578	*P. miyabeana* MT317303	*P. poissonii* NC024543	*P. secundiflora* MT317261
NADH dehydrogenase	*ndhA*, *ndhB* (2), *ndhC*, *ndhD*, *ndhE*, *ndhF*, *ndhG*, *ndhH*, *ndhI*, *ndhJ*, *ndhK*				
Photosystem I	*psaA*, *psaB*, *psaC*, *psaI*, *psaJ*				
Photosystem II	*psbA*, *psbB*, *psbC*, *psbD*, *psbE*, *psbF*, *psbH*, *psbI*, *psbJ*, *psbK*, *psbL*, *psbM*, *psbN*, *psbT*, *psbZ*				
PS I assembly factor		*pafI*, *pafII*	*pafI*, *pafII*		*pafI*, *pafII*
Cytochrome b/f complex	*petA*, *petB*, *petD*, *petG*, *petL*, *petN*				
ATP synthase	*atpA*, *atpB*, *atpE*, *atpF*, *atpH*, *atpI*				
Rubisco	*rbcL*				
Ribosomal RNA genes	*rrn4.5(2)*, *rrn5* (2), *rrn16* (2), *rrn23* (2)				
Transfer RNA genes	*trnA‐UGC* (2), *trnC‐GCA*, *trnD‐GUC*, *trnE‐UUC*, *trnF‐GAA*, *trnfM‐CAU*, *trnG‐UCC*, *trnH‐GUG*, *trnI‐CAU* (2), *trnI‐GAU* (2), *trnK‐UUU*, *trnL‐CAA* (2), *trnL‐UAA*, *trnL‐UAG*, *trnN‐GUU* (2), *trnP‐UGG*, *trnQ‐UUG*, *trnR‐ACG* (2), *trnR‐UCU*, *trnS‐GCU*, *trnS‐GGA*, *trnS‐UGA*, *trnT‐UGU*, *trnV‐GAC* (2), *trnV‐UAC*, *trnW‐CCA*, *trnY‐GUA*				
	*trnG‐GCC*, *trnM‐CAU*, *trnT‐GGU*	*trnM‐CAU*, *trnT‐GGU*	*trnM‐CAU*, *trnT‐GGU*	*trnM‐CAU* (2), *trnP‐GGG*, *trnT‐GGU* (2)	*trnM‐CAU*, *trnT‐GGU*
Small subunit of ribosome	*rps2*, *rps3*, *rps4*, *rps7* (2), *rps8*, *rps11*, *rps12* (2), *rps14*, *rps15*, *rps16*, *rps18*, *rps19*				
Large subunit of ribosome	*rpl2* (2), *rpl14*, *rpl16*, *rpl20*, *rpl22*, *rpl23* (2), *rpl32*, *rpl33*, *rpl36*				
RNA polymerase subunits	*rpoA*, *rpoB*, *rpoC1*, *rpoC2*				
Other genes	*matK*, *cemA*, *accD*, *ccsA*, *clpP*				
Genes of unknown function	*ycf1*, *ycf2* (2), *ycf3*, *ycf4*	*ycf1*, *ycf2* (2), *ycf15* (2)	*ycf1*, *ycf2* (2), *ycf15* (2)	*ycf1*, *ycf2* (2), *ycf3*, *ycf4*, *ycf15* (2), *ycf68* (2), *orf42* (2), *orf188*	*ycf1*, *ycf2* (2), *ycf15* (2)
Pseudogene	*infA*			*infA*	

*Note*: (2): gene with two copies.

### 3.2 Repeat sequences and SSRs analysis

**FIGURE 2:**
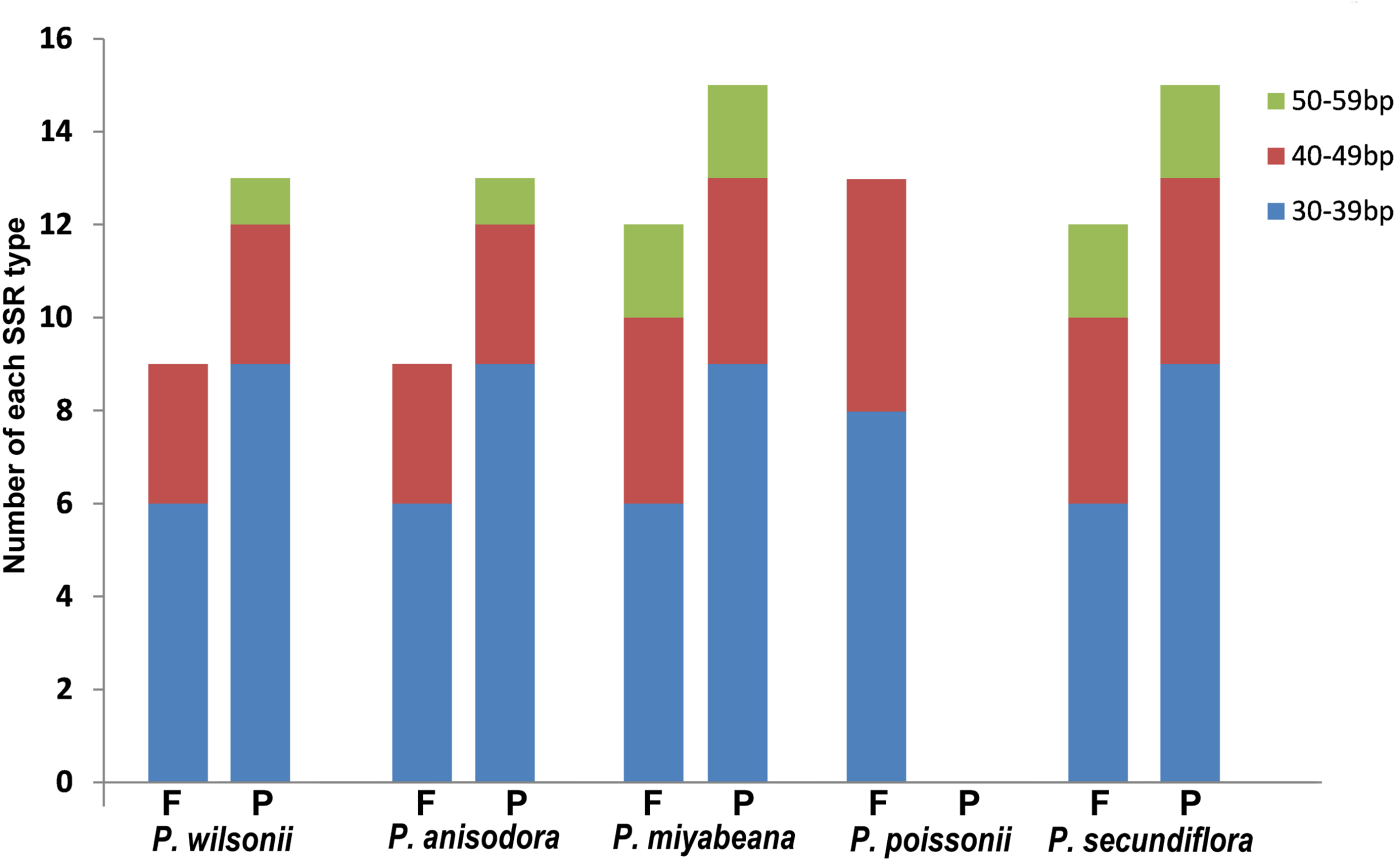
Types and numbers of large repeat sequences in the chloroplast genomes of five *Primula* species. F and P represent forward and palindromic repeats, respectively.

Except for the largest repeat in each genome (IR regions), a total of 111 repeat pairs no more than 60 bp in length were identified in the five *Primula* chloroplast genomes. Only two types of repeat sequences, forward and palindromic repeats, were detected (Figure 2). There were 22 (nine forward, 13 palindrome), 22 (nine forward, 13 palindrome), 27 (12 forward, 15 palindrome), 13 (13 forward), and 27 (12 forward, 15 palindrome) repeats in *P. wilsonii*, *P. anisodora*, *P. miyabeana*, *P. poissonii*, and *P. secundiflora*, respectively. Among them, the chloroplast genomes of *P. miyabeana* and *P. secundiflora* had the largest number of repeats, whereas *P. poissonii* had the fewest. The results indicated that forward and palindrome repeats occupied almost the same proportion. In these five genomes, the length of the repeats was mainly in the range of 30–39 bp, with a percentage of 61.26% (68 of 111 repeats), followed by 40–49 bp, contributing 29.73% (33 of 111 repeats).

A total of 38 microsatellites in the *P. wilsonii* chloroplast genome were detected, including 28 mono‐, five di‐, two tri‐ and three tetra‐nucleotide repeats. In *P. poissonii*, 38 SSRs loci were detected, consisting of 27 mono‐, six di‐, two tri‐ and three tetra‐nucleotide repeats, respectively (Table 3). Moreover, 41 SSRs loci were spotted in *P. secundiflora*, and the numbers of different types are listed in Table 3. Here, we found that the one‐base SSRs loci of *P. wilsonii*, *P. miyabeana* and *P. poissonii* were only A/T repeats. The dinucleotide repeats were AT/TA in all the five *Primula* species. It is shown that the trinucleotide repeats were AAT/ATT in *P. wilsonii*, *P. anisodora*, *P. miyabeana*, and *P. poissonii*, but they were not present in *P. secundiflora*. The tetra‐nucleotide repeats AAAT/ATTT were present in all the *Primula* species. However, the AAAG/CTTT and AATT repeats only appeared in *P. secundiflora*. Among these SSRs loci, 30 (78.95%) were in the LSC region, six (15.79%) were in SSC region, and two (5.26%) were in the IR region of the *P. wilsonii* chloroplast genome.

**TABLE 3:** Types and numbers of simple sequence repeats in the chloroplast genomes of five *Primula* species

SSRs type	Repeat unit	*P. wilsonii*	*P. anisodora*	*P. miyabeana*	*P. poissonii*	*P. secundiflora*
Mono‐	A/T	28	24	30	27	30
	C/G	–	1	–	–	1
Di‐	AT/TA	5	5	5	6	5
Tri‐	AAT/ATT	2	2	2	2	–
Tetra‐	AAAT/ATTT	3	3	3	3	2
	AAAG/CTTT	–	–	–	–	1
	AATT/AATT	–	–	–	–	1
Penta‐	AAATC/ATTTG	–	–	–	–	1
Total		38	35	40	38	41

### 3.3 Sequence divergence

**FIGURE 3:**
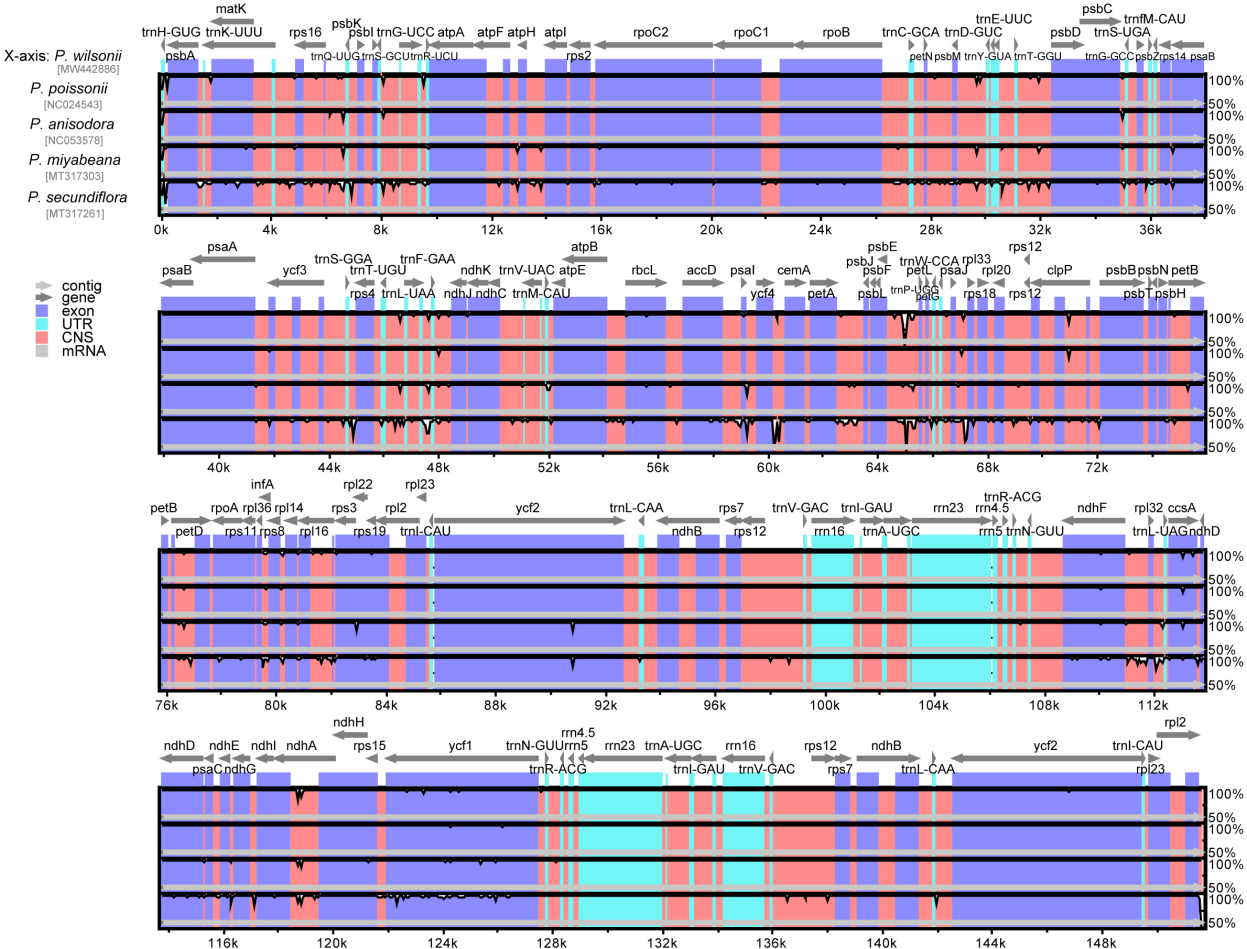
Comparison of the complete chloroplast genome of five *Primula* species, with *P. wilsonii* as a reference. Gray arrows and thick black lines above the alignment indicate gene orientation. The y‐axis represents the percentage identity ranging from 50% to 100%. The acronym CNS represents conserved noncoding sequences.

**FIGURE 4:**
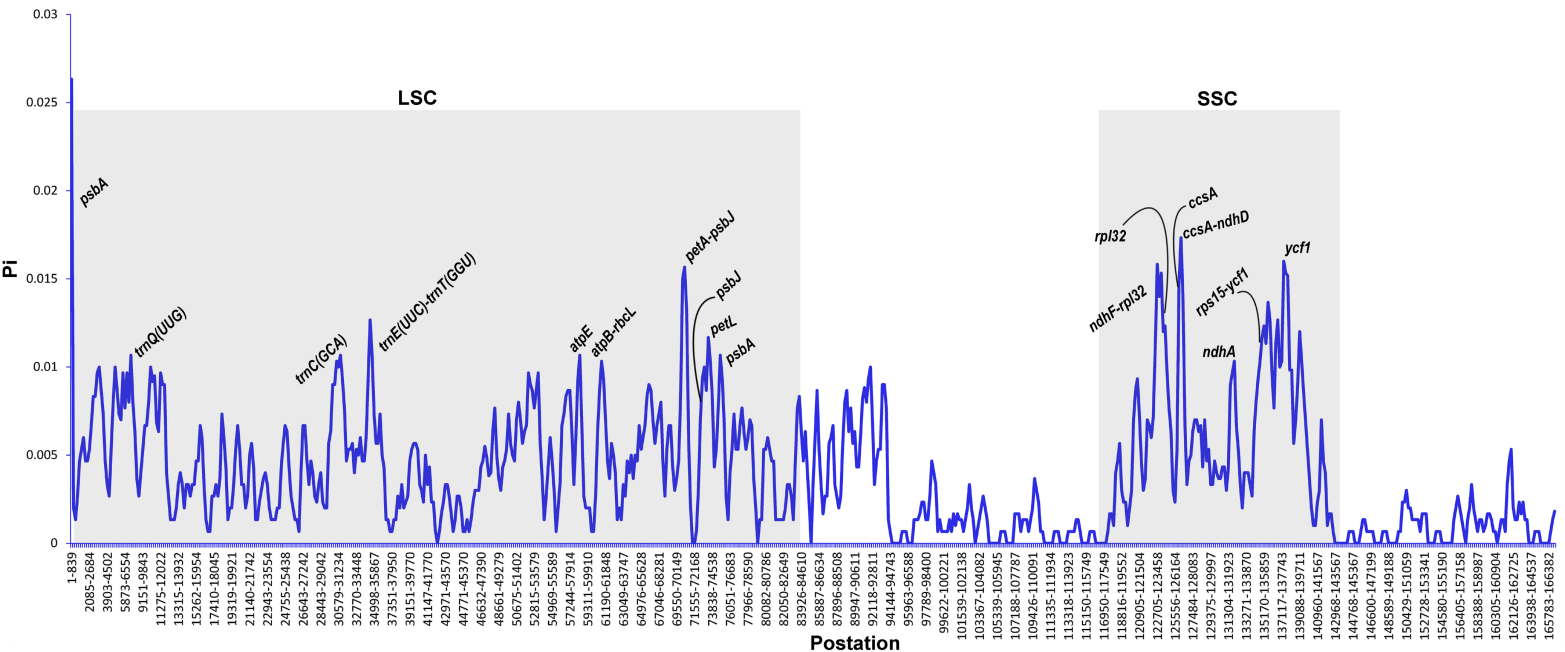
Sliding window analysis of the whole chloroplast genomes of five *Primula* species. The nucleotide variability of the five *Primula* species was assessed by DnaSP with window length of 600 bp and step size of 200 bp. The X‐axis represents the regions of chloroplast genome and Y‐axis represents nucleotide diversity for each window. The 17 hypervariable regions with pi >0.01 were labeled in the figure.

The *Primula* chloroplast genomes exhibited moderate sequence divergence. Furthermore, the results showed that the sequence of coding regions and the two IR regions were significantly more conserved than that of LSC and SSC regions. In the coding regions, most genes were relatively conserved, except for *matK*, *rpl22*, *ndhF*, and *ycf1*. In contrast, the intergenic regions were shown to be highly divergent (Figure 3). Then, we found that the variation level of DNA polymorphism was from 0.00067 to 0.02633. The greatest differences among the five *Primula* species were located in the two SC regions, while IR regions were the least different. About 17 hypervariable regions were discovered with a nucleotide diversity (Pi) value that was greater than 0.01 (Figure 4). Some relatively high Pi value were detected in CDS, such as *psbA* (0.02633), *ycf1* (0.01533), *psaJ* (0.01333), *rpl32* (0.01233), *petL* (0.01167), and *ndhA* (0.01033). Consistently, the gene *ycf1* exhibited higher diversity and showed abundant variation. In addition, we revealed five highly divergent regions among noncoding regions, including *ccsA‐ndhD* (0.01733), *ndhF‐rpl32* (0.01583), *petA‐psbJ* (0.01567), *trnE*(UUC)‐*trnT*(GGU) (0.01267), and *rps15‐ycf1* (0.01167). These mutational hotspots can serve as potential loci when developing novel DNA barcodes for plant classification within the genus *Primula*.

### 3.4 IR expansion and contraction analyses

**FIGURE 5:**
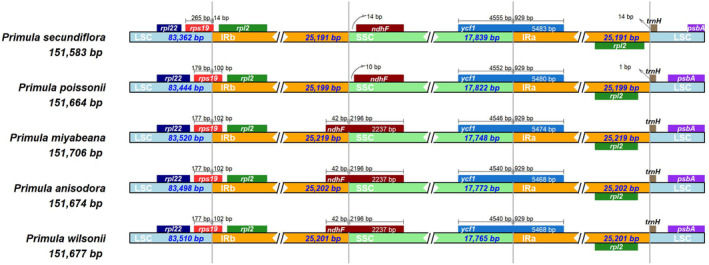
Comparisons of the LSC, IR, and SSC boundary regions among the five *Primula* chloroplast genomes. Genes at the IR/SC borders are denoted by the colored boxes. The numbers within the boxes represent the distances from the boundaries.

The IR/SC junction regions showed slight differences in gene content and gene order. As shown in Figure 5, genes *rps19*/*rpl2*, *ndhF*, *ycf1*, and *rpl2*/*trnH* were present at the junction of the LSC/IRb, IRb/SSC, SSC/Ira, and IRa/LSC borders, respectively. The LSC/IRb boundary was located in the *rps19* region, which crossed into the IRb region in all the five chloroplast genomes, resulting in a variable expansion (14–102 bp) of IRb region toward the *rps19* gene. The *ndhF* gene was entirely located in the SSC region in the chloroplast genomes of *P. secundiflora* and *P. poissonii*, whereas the IRb region extended 42 bp into the *ndhF* gene in all the other *Primula* chloroplast genomes. The SSC/IRa junction was situated in the *ycf1* coding region, which crossed into the IRa region in all the five chloroplast genomes. However, the length of *ycf1* in the IRa region varied from 5468 to 5483 bp, indicating the dynamic variation of the SSC/IRa junctions. The gene *trnH* was located in LSC, 0–14 bp away from the IRa/LSC border (Figure 5). Taken together, these data indicate that the contractions and expansions of the IR regions exhibited relatively stable patterns within these *Primula* species, with slight variations.

### 3.5 Phylogenetic analysis

**FIGURE 6:**
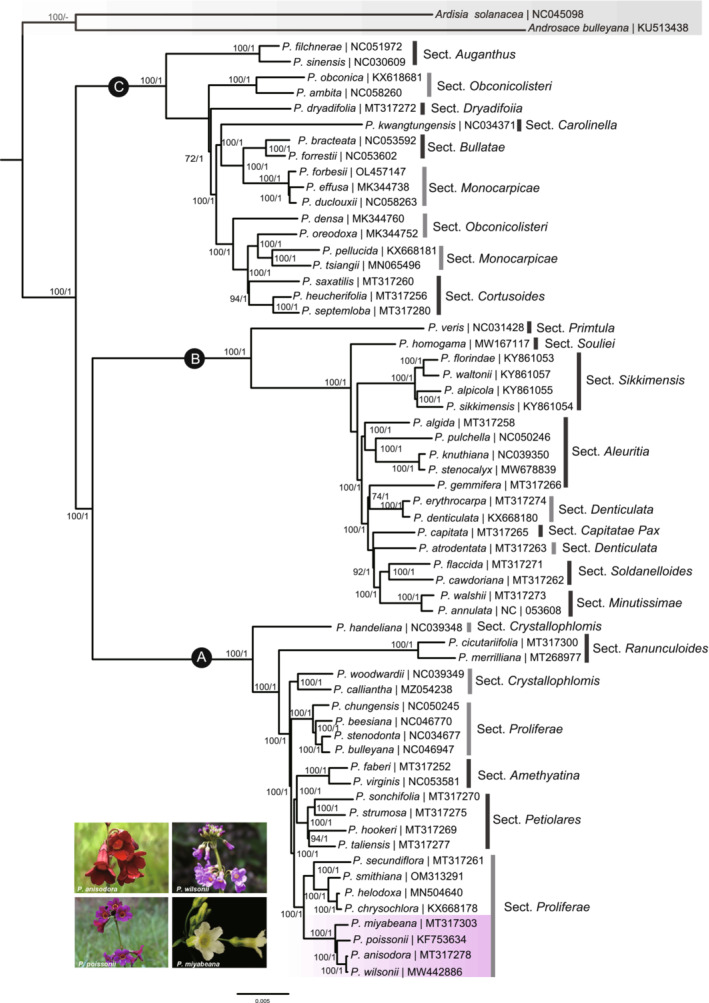
Maximum likelihood (ML) and Bayesian inference (BI) trees of *Primula* species based on chloroplast genomes. Numbers associated with nodes indicated bootstrap support values and Bayesian posterior probabilities. The GenBank accession numbers were displayed following each species. Outgroups and *P. poissonii* complex are highlighted with gray and purple shadings, respectively.

Our sampling represented 20 of 24 recognized sections of the genus *Primula* in China (Hu, 1990). The selected models for whole chloroplast genomes and 66 shared CDS datasets used for BI were GTR+G model and TVM+I+G model, respectively. The different datasets basically produced congruent phylogenetic trees with high support values (Figure 6 and Figure S1). The phylogenetic tree identified that all the samples in the genus *Primula* could be divided into three major clades (Figure 6). Clade A included Sects. *Crystallophlomis*, *Ranunculoides*, *Amethyatina*, *Petiolares*, and *Proliferae*. Clade B contained Sects. *Primtula*, *Souliei*, *Sikkimensis*, *Aleuritia*, *Denticulata*, *Capitatae*, *Soldanelloides*, and *Minutissimae*. Clade C combined Sects. *Auganthus*, *Obconicolisteri*, *Dryadifoiia*, *Carolinella*, *Bullatae*, *Monocarpicae*, and *Cortusoides* species. Our results found that several sections were not monophyletic groups, such as Sects. *Monocarpicae*, *Crystallophlomis*, *Obconicolisteri*, *Denticulata*, and *Proliferae*. It is worth noting that *P. wilsonii* was closest to *P. anisodora* with very high support value in Sect. *Proliferae*. The *P. poissonii* complex was further confirmed, which included *P. wilsonii*, *P. anisodora*, *P. poissonii*, and *P. miyabeana*. However, the monophyly of Sect. *Proliferae* suggested in previous studies was not supported here (Yan et al., 2015).

## 4 DISCUSSION

### 4.1 The general characteristics of *Primula* chloroplast genomes

As with most angiosperms, the chloroplast genomes were conserved in *Primula* species, with similar GC content and typical quadripartite structures, including small and large single copy (SSC and LSC) regions separated by two inverted repeats (IRs) regions (Xin et al., 2019). However, gene loss was found here. The *infA* gene, which encodes translation initiation factor 1 (Wicke et al., 2011), was present in the chloroplast genome of *P. poissonii* and *P. wilsonii*, but was not present in the related *Primula* chloroplast genomes in our study. Additionally, these findings are consistent with the results of some *Primula* species and other groups in angiosperm chloroplast genomes in previous studies (Gichira et al., 2017; Ren et al., 2018). The *ycf15* gene was absent in the chloroplast genomes of *P. wilsonii*. Similar events have also occurred in other angiosperm plastid genomes, such as *Colchicum* genus (Nguyen et al., 2015). *ycf15* was located in the IR region and was highly conserved (Liu, 2018), and the function of the *ycf15* gene remained unclear and needed to be further investigated. The non‐presence of *ycf15* we found here might be a valuable molecular marker to separate *P. wilsonii* from *P. poissonii*, which is morphologically similar to *P. wilsonii*. Both of the two species are perennial herbs with candelabra inflorescence and purple flowers, so some scholars have argued that *P. wilsonii* should be merged into *P. poissonii* or treated as a subspecies of *P. poissonii*. Here we suggest that the missing *ycf15* gene in the *P. wilsonii* chloroplast genome could be extremely useful for distinguishing the two confusing species at the molecular level.

### 4.2 The evolution of the chloroplast genomes in *Primula*

Inverted repeats regions are highly conserved in most angiosperm chloroplast genomes. However, the contraction and expansion of IR regions are not rare (Kim & Lee, 2004). In this study, gene orders at the boundaries of SC/IR regions were the same among these five chloroplast genomes of *Primula*. However, the accurate positions of the genes at the SC/IR borders were slightly varied, such as the genes *rps19*, *ndhF*, *ycf1*, *rpl2*, and *trnH* (Ren et al., 2018). In addition, some genes normally located in the SC region, such as *ndhF*, had moved to IR region due to the expansion of the IR region. It was reported that the chloroplast genomes' size, the LSC/SSC length, the gene numbers and pseudogene origination could vary among different species due to the expansion and contraction of IR regions (Menezes et al., 2018; Saina et al., 2018). Moreover, the loss of IR regions has been occasionally detected in some taxa (Yi et al., 2013). This could be the reason that the chloroplast genome size of *P. miyabeana* was the largest among the five *Primula* species with the longest IR region, and the chloroplast genome size of *P. secundiflora* was the smallest with the shortest IR region. Furthermore, a large number of studies also confirmed that the length of IR region greatly affected the chloroplast genome size (Ren et al., 2021; Sun et al., 2017).

More genomic resources are needed to deeply investigate the phylogeny, biogeography, genetics, and heterostyly evolution of *Primula* species on account of the ornamental and reproductive importance. In addition, considering that *P. wilsonii* is a PSESP, we need more genetic information for the conservation of germplasm resources. The numbers and distributions of repeat sequences, especially large repeats that are longer than 20 and 60 bp, may play important roles in the arrangement and recombination of the plastid genome (Guisinger et al., 2011; Pombert et al., 2006). A total of 111 repeats were detected in the five *Primula* chloroplast genomes. All the repeat sequences appeared to be shorter than 60 bp in length. These findings are consistent with the results in other *Primula* species (Ren et al., 2018; Xu et al., 2020), but not in agreement with the results of some other angiosperm plants, such as herbaceous *Alpinia* species (Li et al., 2020) or woody *Aquilaria* species (Ren et al., 2021). Our study detected very high levels of polymorphism in the large repeat sequences among the five *Primula* species in terms of both the types and numbers. Therefore, these large repeats might be an informative source for developing genetic markers for population genetics and phylogenetic constructions of *Primula* (Hishamuddin et al., 2020). SSRs markers are valuable genetic resources for phylogenetic investigations, population genetics assessment, and species discrimination due to their abundant polymorphism and codominant inheritance (Gulzar et al., 2018; Ren et al., 2021). The SSRs markers detected here were mostly A/T mononucleotide repeats (28/38), similar to the results of other *Primula* species (Ren et al., 2018) and some other angiosperm species (Shen et al., 2020; Wang et al., 2021). The vast majority of SSRs loci were in SC regions (78.95% in LSC regions and 15.79% in SSC regions), yet few of them were present in IR regions. Moderate sequence divergence with greater variability in the SC region of *Primula* chloroplast genomes was displayed, which corresponded with previous studies (such as, Zhu et al., 2016). Since the hypervariable regions of the chloroplast genome are useful for phylogenetic construction, population genetics, and DNA barcoding, the 17 highly polymorphic loci and the SSRs markers found in our study could serve as potential genetic markers for further phylogenetic and biogeographic analyses and population genetics analysis of *Primula* species.

### 4.3 Phylogenetic relationships of Chinese *Primula*

A total of 60 species representing 20 of 24 sections in Chinese *Primula* were sampled in our phylogenetic construction using chloroplast genome sequences based on ML and BI methods. Three major clades of *Primula* were detected with high internal support in this study, which was in accordance with previous studies (Liu, 2018; Xu et al., 2020; Yan et al., 2015). Several sections did not exhibit monophyletic taxa, such as Sects. *Monocarpicae*, *Crystallophlomis*, *Obconicolisteri*, *Denticulata*, and *Proliferae*, which were partly or entirely confirmed by the previous viewpoints (Liu, 2018; Xu et al., 2020; Yan et al., 2015). A decision on the monophyly of Sect. *Proliferae* requires additional consideration. It has been treated as a monophyletic group based on the concatenation of ITS, *matK* and *rbcL* sequences (Xu et al., 2020; Yan et al., 2015). However, the chloroplast transcripts and protein‐coding sequences from chloroplast genomes analyses strengthen the assumption that Sects. *Amethyatina* and *Petiolares* species are nested within Sect. *Proliferae* (Li, 2022; Liu, 2018). This assumption is additionally supported by the results based on the whole chloroplast genome and protein‐coding sequences data analysis in our investigation. This is corroborated by morphological traits such as an umbel with multiple flowers, campanulate calyx, and globose capsule. The conflicting phylogenetic diagnoses of nuclear and chloroplast sequences are common in plants (Degna & Rosenberg, 2009). The adaptive radiation caused by heterostyly, polyploidization, and natural hybridization, or gene introgression might complicate the phylogenetic relationships under *Primula* (De Vos et al., 2014; Guggisberg et al., 2009; Ma et al., 2014; Xie, Zhao, et al., 2017; Xie, Zhu, et al., 2017). Furthermore, chloroplast capture through hybridization may occur in these sections, which has been documented in plants and the genus *Primula* (Casazza et al., 2012; Yi et al., 2015). This would explain why quite a few sections in *Primula* did not belong to monophyletic group according to morphological characters.

*Primula wilsonii*, together with *P. poissonii*, *P. anisodora*, and *P. miyabeana* (endemic to Taiwan) formed to *P. poissonii* complex, which was one of the taxonomically challenging groups in Sect. *Proliferae*. The close relationship of these species has been revealed in previous studies, and *P. wilsonii* was closest to *P. miyabeana* based on *rbcL* + *matK* + ITS sequences, with low support (Yan et al., 2015). However, the closest relative species was *P. anisodora* with very high support based on chloroplast genomic sequences in this study. Therefore, we suggest that the phylogenetic relationships between *Primula* species need to be further studied based on more genetic information, especially at the genomic level, and we may come to the conclusion that chloroplast genomes sequences could provide a valuable resource for phylogenetic constructing of *Primula*.

## 5 CONCLUSIONS

This study compared the basic characteristics of the chloroplast genomes from five Chinese *Primula* species. We assessed the variation and IR boundaries evolution among these species. Furthermore, we constructed the phylogenetic relationships of the genus *Primula* covering a wide range of samples based on their chloroplast genomic sequences. In addition, we determined the conserved and variable regions in the chloroplast genomes. The large repeat sequences, SSRs loci, and 17 hypervariable regions were detected here, which could be used for population genetics and phylogenetic analysis in the future. Three major clades in *Primula* were confirmed, yet the sections were not in accordance with morphological traits, reflecting in the non‐monophyletic nature of several sections. Therefore, we suggest that chloroplast genomes provide useful genetic and evolutionary information for studies on the phylogeny and population genetics of *Primula* species.

## AUTHOR CONTRIBUTIONS

**YanPing Xie:** Conceptualization (equal); data curation (equal); formal analysis (equal); investigation (equal); methodology (equal); project administration (equal); resources (equal); software (equal); supervision (equal); validation (equal); visualization (equal); writing – original draft (equal); writing – review and editing (equal). **GangGang Yang**: Conceptualization (equal); data curation (equal); formal analysis (equal); investigation (equal); methodology (equal); resources (equal); validation (equal); visualization (equal). **Chan Zhang:** Data curation (equal); formal analysis (equal); investigation (equal); methodology (equal); resources (equal); validation (equal); visualization (equal); writing – original draft (equal). **XingWang Zhang:** Conceptualization (equal); data curation (equal); formal analysis (equal); investigation (equal); methodology (equal); project administration (equal); resources (equal); software (equal); supervision (equal); visualization (equal); writing – review and editing (equal). **XianFeng Jiang:** Conceptualization (equal); data curation (equal); formal analysis (equal); investigation (equal); methodology (equal); project administration (equal); resources (equal); software (equal); supervision (equal); validation (equal); visualization (equal); writing – review and editing (equal).

## CONFLICT OF INTEREST

The authors declare that there are no conflicts of interest regarding publication of this paper.

## Supporting information


Figure S1.

## Data Availability

The original sequencing data have been submitted to the NCBI database and received GenBank accession numbers MW442886 (*P. wilsonii*). The data used in this study are already entirely in the public domain (https://www.ncbi.nlm.nih.gov). The GenBank accession numbers of all the species used for phylogenetic analysis are displayed in Figure 6 and Figure S1.
