# Supplementary material for: Comparative analysis of chloroplast genomes of endangered heterostylous species Primula wilsonii and its closely related species
Source: Ecol Evol. 2023 Jan 16;13(1):e9730. doi: 10.1002/ece3.9730 (PMC9842877; doi:10.1002/ece3.9730)
Supplement: Supplementary file 1 — Figure S1. [file ECE3-13-e9730-s001.docx]

**SUPPORTING INFORMATION**


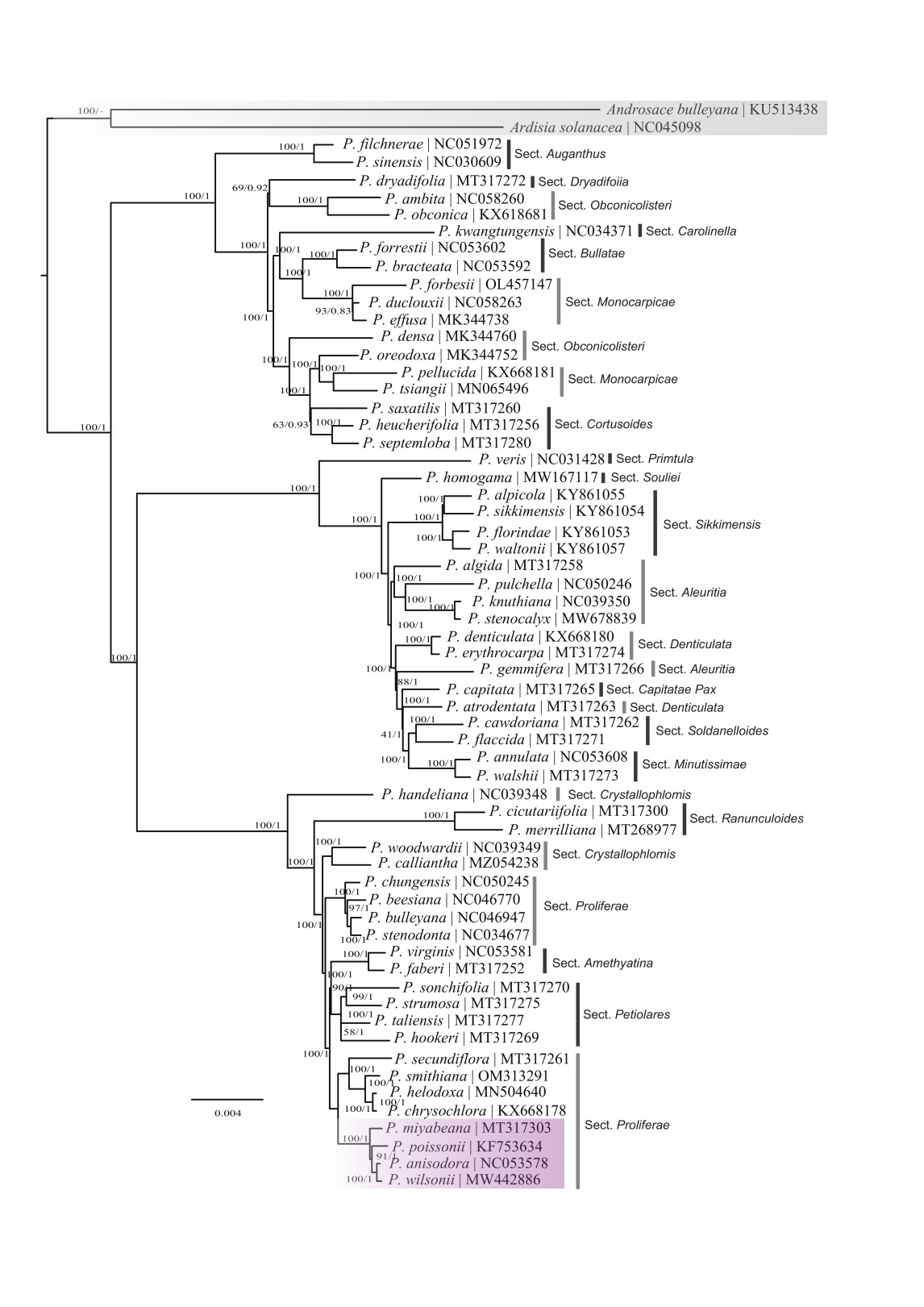


**Figure S1.** Maximum likelihood (ML) and Bayesian inference (BI) trees of *Primula* species based on 66 shared CDS. Numbers associated with nodes indicated Bootstrap support values and Bayesian posterior probabilities. The GenBank accession numbers were displayed following each species. Outgroups and *P. poissonii* complex are highlighted with gray and purple shadings, respectively.
